# Case Report: Cerebral Revascularization in a Child With Mucopolysaccharidosis Type I

**DOI:** 10.3389/fped.2021.606905

**Published:** 2021-06-10

**Authors:** Nathan Grant, J. Michael Taylor, Zach Plummer, Kasiani Myers, Thomas Burrow, Lori Luchtman-Jones, Anna Byars, Adrienne Hammill, Katie Wusick, Edward Smith, James Leach, Sudhakar Vadivelu

**Affiliations:** ^1^Division of Pediatric Neurosurgery, Cincinnati Children's Hospital Medical Center, Cincinnati, OH, United States; ^2^Division of Neurology, Cincinnati Children's Hospital Medical Center, Cincinnati, OH, United States; ^3^Division of Hematology – Oncology, Cincinnati Children's Hospital Medical Center, Cincinnati, OH, United States; ^4^Division of Human Genetics, Cincinnati Children's Hospital Medical Center, Cincinnati, OH, United States; ^5^Department of Neurosurgery, Boston Children's Hospital, Boston, MA, United States; ^6^Department of Radiology, Cincinnati Children's Hospital Medical Center, Cincinnati, OH, United States

**Keywords:** mucopolysaccharidosis I, stroke, cerebral arteriopathy, ventriculomegaly, pial synangiosis, cerebral revascularization

## Abstract

Mucopolysaccharidosis (MPS) type I is a rare lysosomal storage disorder caused by an accumulation of glycosaminoglycans (GAGs) resulting in multisystem disease. Neurological morbidity includes hydrocephalus, spinal cord compression, and cognitive decline. While many neurological symptoms have been described, stroke is not a widely-recognized manifestation of MPS I. Accordingly, patients with MPS I are not routinely evaluated for stroke, and there are no guidelines for managing stroke in patients with this disease. We report the case of a child diagnosed with MPS I who presented with overt stroke and repeated neurological symptoms with imaging findings for severe ventriculomegaly, infarction, and bilateral terminal carotid artery stenosis. Direct intracranial pressure evaluation proved negative for hydrocephalus. The patient was subsequently treated with cerebral revascularization and at a 3-year follow-up, the patient reported no further neurological events or new ischemia on cerebral imaging. Cerebral arteriopathy in patients with MPS I may be associated with GAG accumulation within the cerebrovascular system and may predispose patients to recurrent strokes. However, further studies are required to elucidate the etiology of cerebrovascular arteriopathy in the setting of MPS I. Although the natural history of steno-occlusive arteriopathy in patients with MPS I remains unclear, our findings suggest that cerebral revascularization is a safe treatment option that may mitigate the risk of future strokes and should be strongly considered within the overall management guidelines for patients with MPS I.

## Introduction

Mucopolysaccharidosis (MPS) type I is a rare, autosomal recessive, lysosomal storage disorder caused by a deficiency of the enzyme alpha-L-iduronidase with resultant accumulation of glycosaminoglycans (GAGs) within the lysosomes (1, 2). MPS I is classified into two clinical entities based on disease severity: severe [Hurler syndrome (OMIM #607014)] and attenuated [Hurler-Scheie (OMIM #607015) and Scheie syndromes (OMIM #607016)]. Clinical features include coarse facial appearance, corneal clouding, hepatosplenomegaly, hearing loss, hydrocephalus, cardiac valvular disease, airway obstruction, spinal cord compression, dysostosis multiplex, and cognitive decline (2). Without treatment, individuals with severe MPS I may die within the first decade of life, usually from cardiorespiratory failure or progressive neurological disease (2, 3). Currently, short-term administration of enzyme replacement therapy (ERT) in combination with hematopoietic stem cell transplantation (HSCT) is recommended for patients with severe MPS I and is associated with improved mortality and engraftment rates, reduced urinary GAG excretions, regression of cerebral ventriculomegaly, and stabilization of cardiac ventricular dysfunction (4–7). ERT, however, does not cross the blood-brain barrier in sufficient quantities to address neurological disease (2, 8, 9). Accordingly, the natural history of MPS I may be characterized by progressive neurological morbidity (2, 8–11).

Stroke is not a widely recognized manifestation of MPS I (2, 12, 13). Accordingly, patients with MPS I are not routinely screened for stroke and there are no established guidelines for managing stroke in this population. We report the first case of a 17-month-old male with MPS I who developed an acute ischemic stroke secondary to progressive multifocal cerebral arteriopathy. Cerebral revascularization was performed and long-term follow-up indicated successful engraftment and protection from further stroke. Our findings indicate that cerebral revascularization can be considered an appropriate treatment option for patients with MPS I at risk of stroke.

## Case Description

### History and Presentation

The patient was born at 40 weeks to non-consanguineous parents who are carriers of MPS I. At 9 months, the patient developed dysostosis multiplex, kyphosis, macrocephaly, and coarse facial features. Enzyme analysis revealing abnormally low alpha-L-iduronidase activity and elevated GAG levels confirmed the diagnosis of MPS I (Hurler syndrome) (14). The patient commenced the recommended treatment for MPS I with 8 weeks of ERT followed by HSCT (5, 6). He experienced graft failure following his first HSCT; however, a second round of this combinatorial therapy resulted in successful donor engraftment.

**Figure 1:**
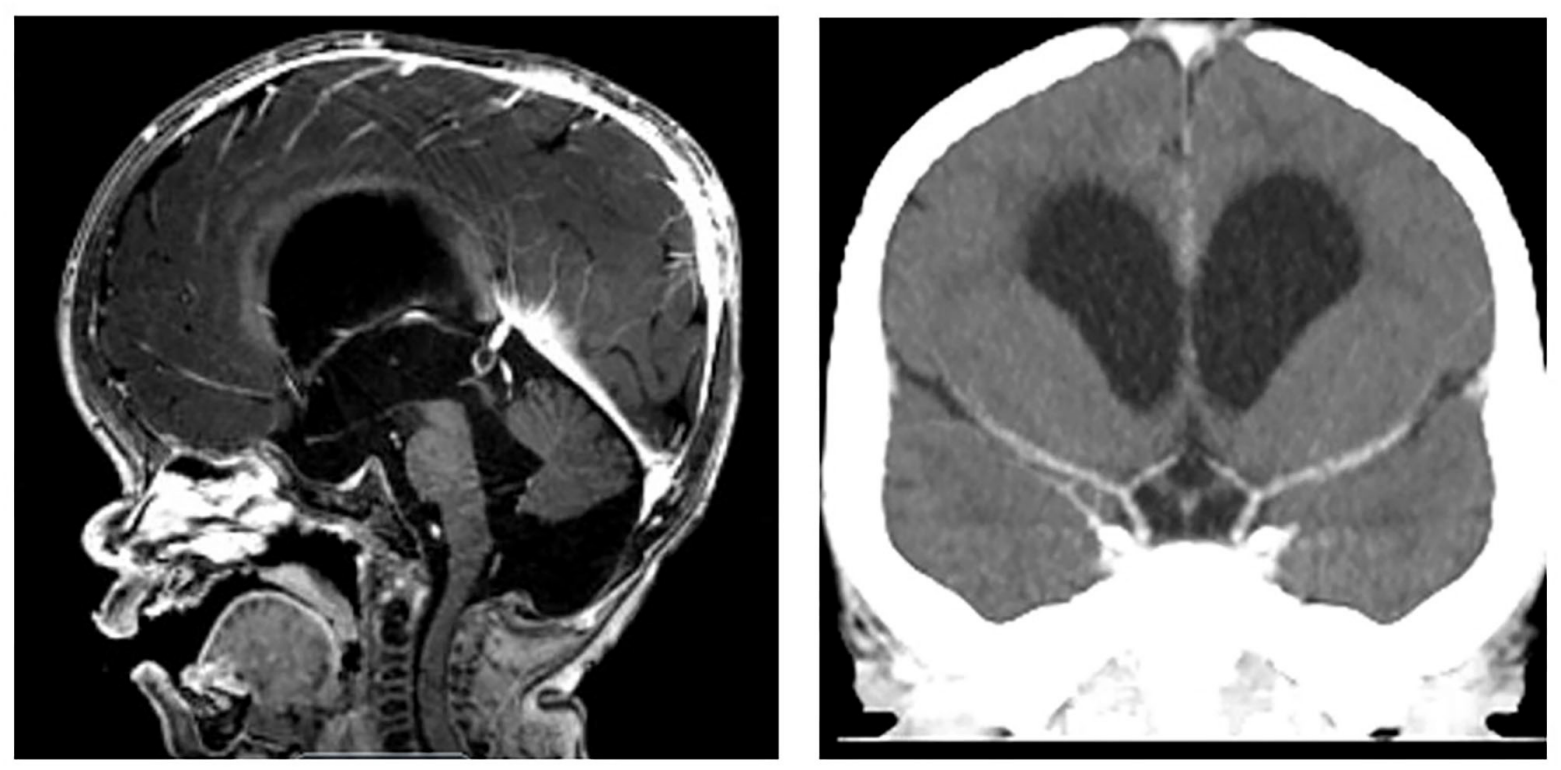
MPS I prior to stroke. **(Left)** MRI Head demonstrating imaging findings of macrocephaly, ventriculomegaly, small foramen magnum, and mild narrowing of cervical spine without cord compression. **(Right)** CT Head with contrast demonstrating bilateral internal carotid termini without stenosis.

Subsequent urinary analysis demonstrated GAG-level reduction consistent with the treatment protocol (4). There was no evidence of graft-versus-host disease, thrombotic microangiopathy, or adverse events related to treatment with ERT and HSCT. Prior to the patient's second HSCT, neuroimaging was obtained for macrocephaly and findings were consistent (15) with MPS I (Figure 1). However, the patient showed no clinical evidence of neurological symptoms at this time.

**Figure 2:**
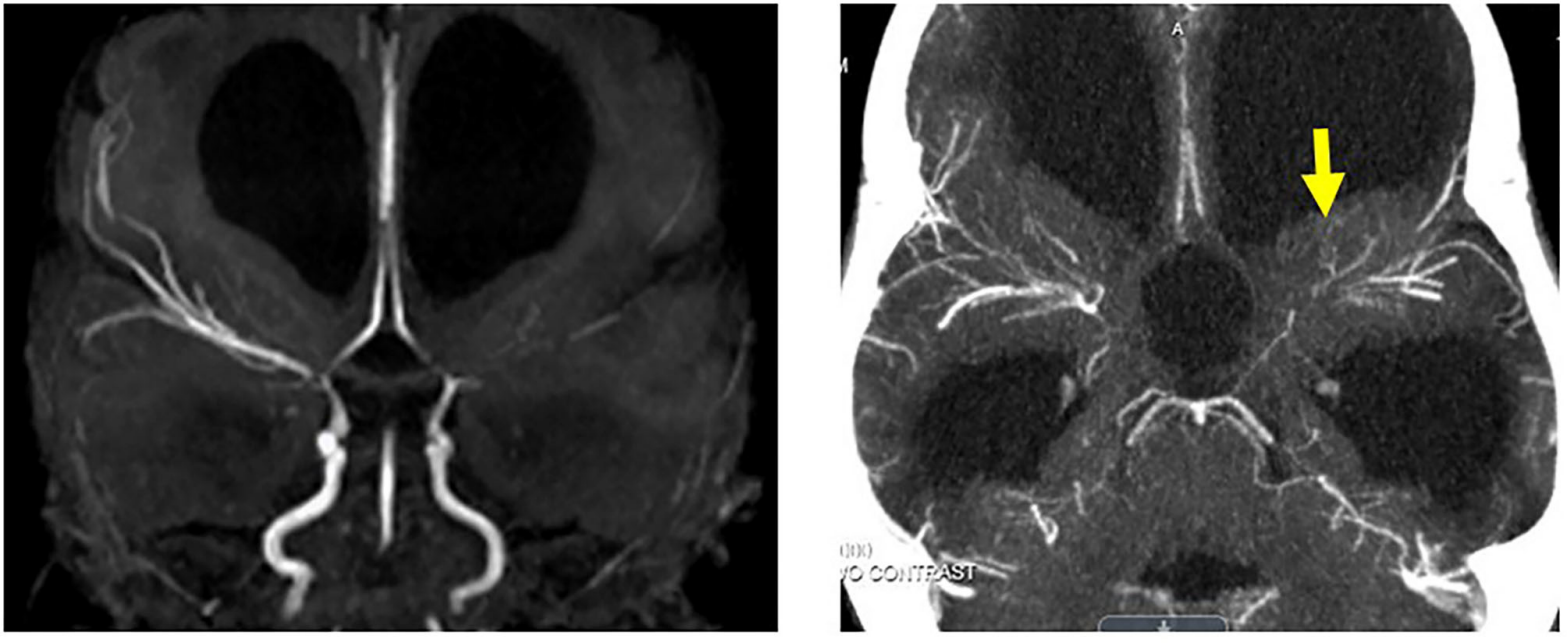
MPS I arteriopathy after left cerebral stroke. **(Left)** MR angiogram 8 weeks after stroke with left MCA occlusion and bilateral ICA termini steno-occlusive disease. **(Right)** New left arm weakness episodes 3 months after overt stroke with persisting bilateral steno-occlusive disease and adjacent irregular collaterals identified on CT angiogram (yellow arrow).

One month after his second HSCT, at 17 months of age, the patient reported new right-sided weakness, emesis, and a decrease in appetite that were concerning for stroke. Brain MRI revealed an acute left middle cerebral artery (MCA) territory infarct and right cerebral ischemia. MR and CT angiography demonstrated complete absence of blood flow into the distal left MCA segments and bilateral carotid terminus narrowing (Figure 2). The patient was given 81 mg aspirin daily.

**Figure 3:**
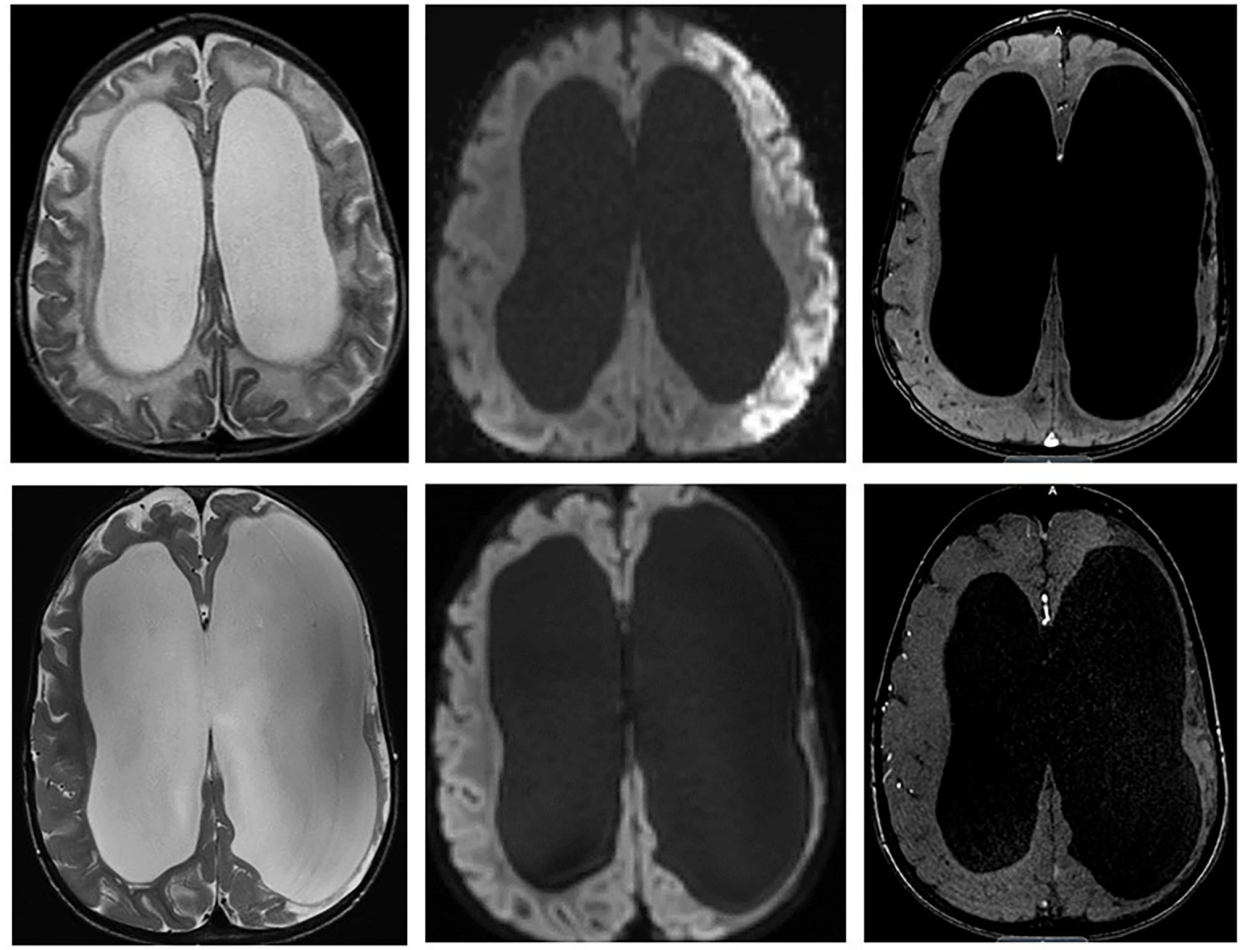
**(Upper)** Initial MRI of left MCA overt stroke identified on T2, diffusion, and MRA. **(Lower)** Two years after right cerebral revascularization demonstrating no new ischemic or infarcted territories and increased right cerebral cortical vascularity.

Serial follow-up MR imaging >6 months post stroke demonstrated continued stroke evolution and bilateral internal carotid artery stenoses (Figure 3, upper panel). Digital subtraction angiography was not performed because of the patient's abdominal aortic aneurysm and renal artery stenosis. Cardiac echocardiograms demonstrated mild mitral regurgitation without evidence of vegetation and EKG, thrombophilia, and platelet function testing were unremarkable. The patient subsequently underwent direct intracranial pressure measurement and therapeutic drainage trials, which revealed normal intracranial pressures without clinical or ventricular improvement. Hydration and hemoglobin status remained stable. Therefore, a diagnosis of bilateral intracranial steno-occlusive arteriopathy was established in the setting of MPS I.

### Surgical Treatment

To our knowledge, there is no literature describing surgical outcomes for steno-occlusive cerebrovascular disease in patients with MPS I. Accordingly, a multidisciplinary review in our Cerebrovascular Center at Cincinnati Children's Hospital and a secondary review with Boston Children's Hospital were convened to discuss treatment options. Given the patient's repetitive presentations of arm weakness and irritability, right steno-occlusive disease, and absence of elevated ICP, we collectively recommended right cerebral revascularization. Left cerebral revascularization was not recommended due to the patient's large-territory, completed stroke.

The patient underwent right-sided indirect revascularization with pial synangiosis and dural inversion, which was well-tolerated without post-operative complication (16, 17). A 2-year postoperative MRI demonstrated increased cortical vascularity without progressive ischemic changes (Figure 3, Lower panel). At a 3-year follow-up, the patient was clinically well with resolution of paresis and without recurrent symptoms.

## Discussion

For the first time, we describe a child with MPS I who presented with an acute ischemic stroke and underlying cerebral arteriopathy. Although strokes have been reported in patients with MPS I (12, 13, 18–20), stroke is not a widely-recognized clinical manifestation of MPS I and there are no reviews or guidelines for managing stroke in patients with this disease (2, 12, 13). This report, to our knowledge, is the first clinical description of the development of progressive cerebral arteriopathy treated via revascularization surgery in a patient with MPS I. Our findings suggest that cerebral revascularization is a safe treatment option that may mitigate future stroke risk and should be considered within the overall management guidelines for patients with MPS I.

### Stroke in MPS I

Neurological manifestations of MPS I include hydrocephalus, spinal cord compression, and cognitive decline (2, 10, 11, 21). Cardiac manifestations include hypertension, arrhythmia, valvular disease, and coronary artery disease (2, 7, 22). Stroke is not a widely recognized manifestation of MPS I, and patients with MPS I are not routinely evaluated for stroke (2, 12, 13). However, a few cases of stroke have been reported in patients with this disease (Table 1) (12, 13, 18–20). A cardioembolic infarction disrupting the left internal carotid artery was the reported cause of stroke in a 41-year-old female with MPS I. After treatment with a recombinant tissue plasminogen activator (rtPA), this patient demonstrated signs of clinical improvement with recanalization of the left internal carotid artery (12). Another child with MPS I presented with an MCA territory occlusion and was treated with 7 days of heparinization, though no neurological changes were observed (13). Along with our current report, these studies indicate that stroke occurs in MPS I; however, it is uncertain whether rtPA therapy and anticoagulants will mitigate the risk of future strokes in patients with this disease. More research into the pathophysiological cause of stroke in MPS I and long-term treatment outcomes can help develop guidelines for managing stroke in this population.

**Table 1:** Stroke reported in patients with mucopolysaccharidosis (MPS) type I.

**Study**	**Year**	**Country**	***N***	**Patient Characteristics**	**MPS I subtype**	**Clinical presentation**	**Imaging: imaging results**	**Diagnosis**	**Treatment**	**Follow-up (time after treatment)**
Belani et al. (18)	1993	United States	1^a^	18-month-old patient (sex not specified)	Hurler syndrome	Intraoperative stroke during Leonard catheter placement	Not specified	Stroke (etiology not specified)	Not specified	Authors report patient showed partial recovery (time not specified)
Souillet et al. (19)	2003	France	1^b^	6-year-old male	Hurler-Scheie syndrome	Not specified	MRI: imaging findings not specified	Stroke (etiology not specified)	Not specified	Not specified
Fujii et al. (12)	2012	Japan	1	41-year-old female	Scheie syndrome	Acute onset dysarthria, right upper limb weakness, right central facial paralysis, mild right hemiparesis	MRI: subtle high signal lesions in left corona radiata and posterior limb of internal capsule MRA: disruption of left internal carotid artery Echocardiogram: mild aortic regurgitation	Stroke from cardioembolic infarction	Intravenous rtPA therapy with 0.6 mg/kg alteplase Commenced treatment with warfarin	Right arm weakness improved, MRA showed recanalization of left internal carotid artery(13 h)No further progression (22 months)
Hill and Preminger (20)	2014	United States	1^c^	9-month-old male	Hurler syndrome	Patent foramen ovale, new onset seizures	MRI: infarct affecting the left MCA territory	Stroke from paradoxical embolism	Percutaneous device closure of patent foramen ovale	No stroke-like symptoms (~14 months)
Olgac et al. (13)	2018	Turkey	1	3-year-old female	Severity not specified	Left hemiplegia, right-sided central facial paralysis, increased deep tendon reflexes	MRI: restricted diffusion in right temporoparietal lobe MRA: right MCA occlusion Transthoracic echocardiography: moderate mitral and aortic valve regurgitation, high carotid intima-media thickness	Stroke from right MCA occlusion	Low-molecular-weight heparin therapy	No change in neurological condition (7 days)
Grant et al.	Current Report	United States	1	17-month-old male	Hurler syndrome	Right-sided weakness, emesis, decrease in appetite	MRI: left MCA infarction MRA: bilateral terminal internal carotid artery stenosis	Stroke from steno-occlusive cerebral arteriopathy	Right indirect cerebral revascularization	No complications during surgeryIncreased cortical vascularity, no new ischemic changes, near total resolution of right-sided weakness (3 years)

*ERT, enzyme replacement therapy; MCA, middle cerebral artery; MPS, mucopolysaccharidosis; MRA, magnetic resonance angiography; MRI, magnetic resonance imaging; rtPA, recombinant tissue plasminogen activator*.

a *Reported in a case series of 30 patients with MPS I, II, III, IV, and VI, of whom one patient who had MPS I presented with stroke*.

b *Reported in a chart review of 27 patients with MPS I who collectively received 30 hematopoietic stem cell transplants between 1986 and 2001; 1 patient presented with stroke*.

c *Case series of 2 identical twin boys with MPS I, of whom one presented with stroke*.

### Diagnostic Evaluation and Stroke Management

The patient described in this report was diagnosed with MPS I at 9 months and presented with a stroke at 17 months of age. The patient's symptoms and imaging are indicative of cerebral arteriopathy with bilateral internal carotid artery stenosis and left MCA occlusion. MRI examinations also demonstrated significant cerebral ventriculomegaly. The possibility of hydrocephalus-related vascular compression was evaluated through repeat lumbar punctures and intracranial pressure monitoring; however, all procedures confirmed normal intracranial pressure. The patient's cerebral ventriculomegaly was therefore consistent with *ex vacuo* changes of ischemic stroke rather than hydrocephalus.

Although the patient's stroke in association with bilateral internal carotid artery stenosis resembled moyamoya, we could not perform a conventional digital subtraction cerebral arteriogram in this patient to diagnose moyamoya (23). Nevertheless, the patient exhibited critical stenoses and distal ischemia on MRI with waxing and waning clinical presentation. It is unlikely the serial MRAs overestimated the high degree of steno-occlusive arteriopathy given the patient's stable hydration and hemoglobin status. Furthermore, there was no evidence for cerebral vasculitis or cardiac abnormalities indicative of cardioembolic stroke. Therefore, a multidisciplinary team was convened to discuss treatment options for this patient. Continued clinical surveillance amid repetitive stroke-like presentations was considered of great risk; thus, cerebral revascularization was collectively recommended to mitigate the risk for recurrent stroke.

### Potential Etiology

Our findings are consistent with a steno-occlusive disease, although the exact etiology of cerebral arteriopathy in patients with MPS I remains unclear. GAG accumulation is common in patients with MPS disorders and has been associated with many neurological and cardiac manifestations of MPS I (12, 21, 24). It is possible that an accumulation of GAGs contributed to this patient's cerebral arteriopathy, ultimately resulting in stroke. Proteoglycan imbalance may affect the arterial smooth muscle cell proliferation rate during MPS I elastogenesis (25), which may be like other intracranial steno-occlusive diseases (26). As we cannot demonstrate a molecular pathway for causation here, further studies will be necessary to determine the exact etiology of cerebral arteriopathy and stroke in patients with MPS I.

### Treatment Recommendations and Outcomes

As cerebral arteriopathy predisposes patients to stroke, treatment is needed to mitigate the risk of recurrent strokes in MPS I. Surgical revascularization prevents further ischemic injury by increasing collateral blood flow to areas of insufficient perfusion due to arteriopathy using external circulation as a donor supply (17, 23, 27). Indirect cerebral revascularization is considered safe and effective for revascularizing the pediatric brain and preventing future stroke (17, 27, 28). While revascularization surgery has been documented in one patient with MPS I, this finding is listed in a large cohort study without clinical presentation, imaging results, and postoperative outcomes (29). The lack of MPS I revascularization evidence made management challenging, necessitating multidisciplinary review. Our findings demonstrate that cerebral revascularization is a safe and effective treatment option to mitigate the risk of recurrent stroke in children with MPS I. Cerebral revascularization should be considered within the overall management of this disease.

## Conclusion

This report provides two important new findings. First, we demonstrate that patients with MPS I are at risk of significant overt stroke secondary to cerebral arteriopathy. As symptoms of MPS I typically emerge in early childhood (2, 11), pediatric providers should consider screening for stroke in children with this disease. Second, this is the first clinical description of cerebral revascularization in a patient with MPS I, supported by long-term follow-up that confirmed favorable tolerance of surgery without any transient or neurological stroke events. Further investigation will help establish guidelines for identifying and treating stroke in patients with MPS I.

## Data Availability Statement

The original contributions presented in the study are included in the article/Supplementary Material, further inquiries can be directed to the corresponding author/s.

## Ethics Statement

Ethical review and approval was not required for the study on human participants in accordance with the local legislation and institutional requirements. Written informed consent to participate in this study was provided by the participants' legal guardian/next of kin. Written informed consent was obtained from the minor(s)' legal guardian/next of kin for the publication of any potentially identifiable images or data included in this article. Informed consent was obtained from the patient for being included in this case report.

## Author Contributions

NG collected data, analyzed and interpreted the data, drafted the initial manuscript, reviewed, and revised the manuscript. JT conceptualized and organized the case report, analyzed and interpreted the data, and critically revised the manuscript for important intellectual content. ZP, KM, TB, LL-J, AB, AH, KW, and ES analyzed and interpreted the data and critically revised the manuscript for important intellectual content. JL and SV conceptualized and organized the case report, analyzed and interpreted the data, drafted the initial manuscript, and critically revised the manuscript for important intellectual content. All authors have approved the final manuscript and agree to be accountable for all aspects of the work.

## Conflict of Interest

The authors declare that the research was conducted in the absence of any commercial or financial relationships that could be construed as a potential conflict of interest.

## Abbreviations

ERT: enzyme replacement therapy
GAGs: glycosaminoglycans
HSCT: hematopoietic stem cell transplantation
ICP: intracranial pressure
IONM: intraoperative neurophysiologic monitoring
MCA: middle cerebral artery
MMA: middle meningeal artery
MPS: mucopolysaccharidosis
rtPA: recombinant tissue plasminogen activator
STA: superficial temporal artery.
